# Case report: A 10-year prognosis of neonatal diabetes caused by a novel *INS* gene mutation

**DOI:** 10.3389/fendo.2022.1086785

**Published:** 2023-01-06

**Authors:** Mengting Tian, Yi Feng, Yanyan Liu, Hua Wang

**Affiliations:** ^1^ Department of Pediatrics, West China Second University Hospital, Sichuan University, Chengdu, China; ^2^ Key Laboratory of Birth Defects and Related Disease of Women and Children (Sichuan University) Ministry of Education, Sichuan University, Chengdu, China; ^3^ Prenatal Diagnosis Center, Department of Obstetrics & Gynecologic, West China Second University Hospital, Sichuan University, Chengdu, China

**Keywords:** *INS*, diabetes homozygosity, diabetes treatment, diabetes follow-up, diabetes case study, autosomal recessive inheritance, neonatal diabetes mellitus (NDM)

## Abstract

**Background:**

Neonatal diabetes mellitus (NDM) is a rare form of diabetes. We analyzed a novel insulin gene (*INS*) mutation of a Chinese permanent neonatal diabetes mellitus (PNDM) patient to explore the clinical and genetic characteristics and put forward some opinions on treatment and its long-term management.

**Case description:**

A proband was recruited who was diagnosed with permanent neonatal diabetes on his first day after birth. His clinical and follow-up data were collected for 10 years. All of the family members were given an oral glucose tolerance test. Whole exome sequencing was performed on the proband, and the genomic DNA of family members was used for verification by first-generation Sanger sequencing technology. The pathogenic variant was screened according to the American College of Medical Genetics and Genomics classification guidelines and the clinical phenotype of the patient.

**Diagnostic assessment:**

The proband was diagnosed on the first day after birth, presenting with low birth weight, progressive hyperglycemia, and insulin deficiency. His parents and grandfathers were confirmed to have normal blood sugar levels. A novel homozygous mutation of c.1T>C in the *INS* gene was detected in the proband, located in the initiation codon. The heterozygous mutations were found in four family members, including his mother, father, and grandfathers. With regular insulin injections, long-term regular follow-up, close monitoring of blood glucose, balanced exercise and diet, and psychological and mutual family support, the blood glucose level was well controlled; there were no acute or chronic complications during this decade. The patient’s growth and nervous system development are now no different to those of the same age.

**Conclusion:**

A favorable prognosis is presented for a permanent neonatal diabetes mellitus (PNDM) patient with a novel mutation in the *INS* gene in China. The present findings indicate that the genetic diagnosis, early use of insulin, close monitoring of blood glucose, and psychological and mutual family support for patients with *INS* mutation are necessary for their favorable long-term prognosis.

## 1 Introduction

Neonatal diabetes mellitus (NDM) is a rare disease that develops within 6 months after birth, with an incidence of about 1/90,000 to 1/260,000 live births (1–4). The incidence varies according to family structure and genetic profile in different regions (5–9). It is generally accepted that the incidence of NDM is higher in countries with a higher consanguinity rate.

According to the difference in insulin dependency, NDM can be divided into transient neonatal diabetes mellitus (TNDM) and permanent neonatal diabetes mellitus (PNDM). Most TNDM develops early and can heal by itself in the first year after birth. More than 40 gene mutations can lead to NDM. Children with different mutations have diverse severity complications, with different treatment choices and long-term prognosis. Mutations in the potassium channel genes (*ABCC8* and *KCNJ11*) are considered the most significant causes of PNDM, accounting for about 40–50% of cases. Mutations in the insulin gene (*INS*) are the second most common cause of PNDM (10), accounting for about 30% of cases. Compared with the severe intellectual disability and other complications caused by some gene mutations (*KCNJ11*, *GATA4*, etc.), the complications associated with *INS* mutation are relatively few.

In this report, we introduce a case of NDM caused by *INS* gene mutation; no previous case report of this gene locus mutation has been reported.

## 2 Case description

### 2.1 Clinical study

A 10-year-old patient with PNDM was recruited for this study in 2021. Clinical and follow-up information for the past 10 years was obtained. Blood samples from the proband and his family members were extracted. All family members underwent an oral glucose tolerance test, and the levels of fasting insulin, fasting C-peptide, and insulin antibody (including anti-islet cell antibody, anti-glutamate dehydrogenase antibody, and anti-insulin antibody) were tested.

### 2.2 Genetic sequencing and bioinformatics analysis

Peripheral blood (2–4 mL) was collected in EDTA anticoagulant tubes from the family members and transported to the laboratory in ice packs. Whole exome sequencing (WES) was performed on the proband. Genomic DNA was extracted from whole blood and then fragmented to 150–350 bp using an ultrasonic crusher. The ends of the broken DNA fragments were then filled in; an “A” base was added at the 3′ end so that the DNA fragment was connected to a special linker with a “T” base at the 5′ end. The library was then amplified with adapters, and AMPure XP was used for PCR purification. Exon capture probes were used to target regions in constructed libraries. The product was enriched by PCR amplification, and a NovaSeq 6000 system (Illumina Inc) was used for the sequencing analysis. BWA, Samtools, and Picard software compared reads with the human reference genome GRCh38/HG38. GATK was used for local re-alignment, the repeat sequences were removed, and the mutation was detected. A series of principles were applied to identify pathogenic mutations based on the variant annotations, as follows: (1) Exon region variants and non-synonymous mutation sites were screened. (2) No healthy person was found carrying this mutation in ExAC_EAS, ExAC_ALL, 1000Genomes, or gnomAD databases, or the population carrier rate was less than 5%. (3) The pathogenic mutation sites were evaluated by referencing dbSNP, OMIM, HGMD, ClinVar, and other databases. (4) SIFT, Polyphen2, LRT, MutationTaster, FATHMM, and other software were used for protein function prediction. Pathogenic variants were screened according to the American College of Medical Genetics and Genomics (ACMG) classification guidelines and the clinical phenotype of the patients. Finally, first-generation Sanger sequencing technology was used for verification, and the samples of the family members were also verified.

**Figure 1:**
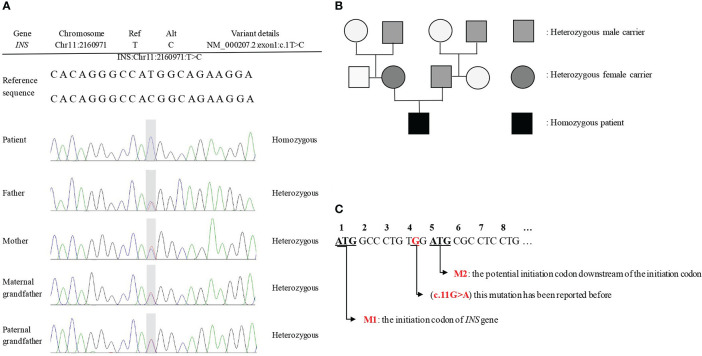
The results of Sanger sequencing and family pedigree. **(A)** The Sanger sequencing showed a novel mutation c. 1T>C (p.M1V) in the *INS* gene. **(B)** The family pedigree. The proband's parents and grandfathers are all heterozygous mutations at the locus, but none of them shows decreased glucose tolerance. **(C)** The transcript of *INS* gene.

The results of Sanger sequencing (**Figure 1A**) showed that a novel homozygous mutation c.1T>C (p.M1V) was found in the *INS* gene of the proband. No pathogenic reports were found in the HGMD and Clinvar databases until now. The family pedigree verification analysis showed that his parents and grandfathers were all heterozygous carriers of the same mutation (**Figures 1A, B**), which supported the evidence (PM2 + PM3). According to ACMG recommendations (11): for the start codon variation, if there is a pathogenic variation upstream of the potential second start codon, the highest level of evidence of start codon variation is PVS1_Moderate. Referring to the amino acid sequence in the transcript (**Figure 1C**), the site where the mutation is located is the initiation codon (M1), and the fourth amino acid downstream of the initiation codon may be the potential initiation codon (M2). In the search, we found that the third amino acid downstream of the initiation codon (c.11G>A) (12) had pathogenic mutations reported in the literature. Therefore, the level of evidence for the start codon variation was PVS1_Moderate. When “chr11-2160971 c.1T>C” was used as the retrieval word in the dbSNP database of common frequency SNPs, the genetic polymorphism with serial number rs757124361 was found; the mutation frequency of this locus is very low. Therefore, the rating of this evidence was moderate (PM2). In summary, the variation chr11-2160971 c.1T>C (p.M1V) (reference genome: GRch38) is to be assessed as a possible pathogenic variation (LP), neonatal diabetes mellitus, autosomal recessive inheritance.

## 3 Diagnostic assessment

**Figure 2:**
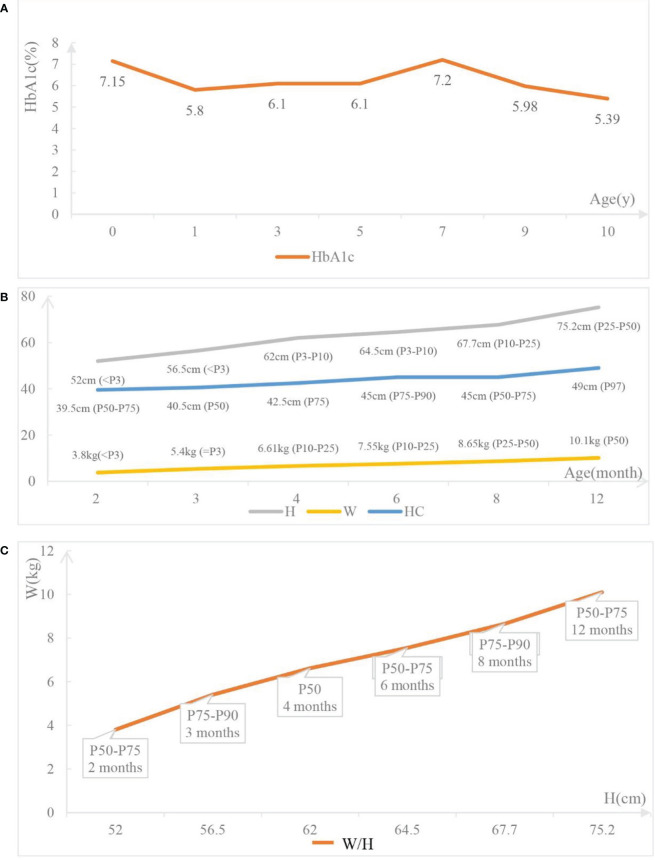
The follow-up data of the patient. **(A)** The hemoglobin Alc (HbA1c) levels of the patient at different time point. **(B)** Growth and development of the patient. H: the height of the patient; W: the weight of the patient; HC: the head circumference of the patient. **(C)** W/H: the weight-for-height of the patient.

The 10-year-old patient was born at 38 + ^5^wk *via* cesarean section delivery on October 18, 2011, with a birth weight of 1.8 kg. The Apgar scores at 1, 5, and 10 minutes were all 10. There is no family history of diabetes, and his parents are not inbred. Furthermore, there was no consanguinity in the five generations before the parents. A progressive increase of blood glucose up to 27.5 mmol/L was found 6 hours after his birth, with low C-peptide (0.03 nmol/L) and insulin (0.7 μIU/mL). Urine ketone body and insulin antibody tests were all negative. Insulin was immediately given subcutaneously (1.1 U/d), and the blood glucose decreased to 3.8 mmol/L. Since genetic testing had not been carried out, we changed to prescribing glibenclamide to control blood glucose, but the control was poor. Eventually, the blood glucose was controlled by successive subcutaneous injections of protamine biosynthetic human insulin (premix 30R), while adjusting the volume of milk given to 120 mL every 4 hours. By adjusting the amount and time of the milk drinking and the insulin injection, the blood glucose was well controlled. The hemoglobin A1c (HbA1c) level was 7.15% when he was discharged 56 days after birth. After discharge, he was fed every 4 hours, and 0.25 U insulin was injected half an hour before each meal. Until he was 2 years old, protamine biosynthetic human insulin (premix 30R) was injected before each meal (5 U/d). Since starting regular meals (from 2 years old), his treatment was changed to an injection of aspartic insulin before meals, increasing from 3 to 7 U per meal year by year until the age of 6. After turning 6, in addition to an injection of aspartic insulin before meals, 5 U/d of detemir insulin was injected before bed, gradually increasing to 6 U/d at present. At the age of 10, 8 U aspartic insulin was injected before each meal (with a fluctuation of 2 U depending on the appetite), and 6 U/d of detemir insulin was injected before bed. During the past 10 years, his blood glucose was checked four times daily and was well controlled. The dosage of insulin given was increased gradually with the increase in body weight, and there were no rapid increases or decreases in the dosage in a short period of time. There were no emergencies related to serious blood glucose fluctuations except hypoglycemia twice. The HbA1c level has been stable for 10 years (**Figure 2A**).

The proband underwent a detailed examination at 10 years old. Physical examination showed no abnormal development and no fat atrophy at the injection site. In addition, his excellent athletic performance is also worth noting. Detailed results are shown in **Table 1**. His family members show normal blood glucose levels, and there is no decrease in glucose tolerance. Diabetes-related examination of family members was carried out simultaneously (**Table 2**).

**Table 1:** Detailed examination results of the patient in his ten years old (2021.11.01).

Item	Result
Height	135 cm (25th centile for corresponding age)
Weight	30.6 kg (25th-50th centile for corresponding age)
BMI	16.79 kg/m^2^ (50th centile for corresponding age)
Blood glucose	6.86 mmol/L
HbA1c	5.39%
C-peptide	0.06 ng/mL
Fasting insulin	65.68 μIU/mL (after insulin injection)
Urinary ketone	Negative
Insulin antibody (ICA, GAD and IAA)	Negative
Liver function	Normal
Kidney function	Normal
Fundus examination	Normal
Peripheral nerve function of lower limbs	Normal
Intelligence quotient (IQ)	106 (tested by Chinese - Wechsler Intelligence Scale for Children)
Physical examination	No abnormal development

**Table 2:** Related examination results of family members (2021.11.01).

	Patient	Mother	Father	Maternal grandfather	Paternal grandfather	Reference range
Age(years)	10	39	38	68	63	/
Body Mass Index (kg/m2)	16.79	20.39	26.36	22.86	25.07	18.5-23.9
Fasting glucose (mmol/l)	6.86	5.1	5.6	/	4.9	3.9-6.1
Two hours post-meal glucose(mmol/l)	/	6.5	6.4	6.4	5.0	3.89-7.8
Fasting insulin(μIU/ml)	168.68*	4.15	12.4	19.48	5.68	3-25
Fasting C-peptide(ng/ml)	0.06	1.42	2.50	6.10 (after diet)	1.01	1.10-4.4
Insulin antibody	Negative	Negative	Negative	Negative	Negative	Negative

*The figure was tested after insulin injection.

## 4 Discussion

Mutations in the *INS* gene can cause various types of diabetes, including neonatal diabetes mellitus, maturity-onset diabetes of the young, and type I diabetes (especially antibody-negative type I diabetes) (10). However, the mutation of the locus (c.1T>C) in our case has not been reported in the literature. Children with different gene or locus mutations have different clinical manifestations and prognosis; therefore, the results of gene testing and case reports of different mutations are extremely important to judge the severity of the disease.

Most published cases show that NDM caused by *INS* mutations is inherited by autosomal dominant inheritance. Only a few cases show that *INS* mutations are inherited by autosomal recessive inheritance. The genetic test shows that the proband has a homozygous mutation at the c.1T>C locus, inherited by autosomal recessive inheritance. Such a mutation at the initiation codon can lead to an abnormal translation process, reduction, or even loss of proinsulin production, but not abnormal insulin structure or endoplasmic reticulum (ER) stress. While the heterozygous mutations in the *INS* gene lead to proinsulin misfolding, which gradually accumulates and causes stress on the ER, leading to severe organelle dysfunction and eventually resulting in apoptosis of the β-cell (13). Compared with patients with *INS* heterozygous mutations, those with homozygous mutations always have a lower birth weight and an earlier age at diagnosis (14, 15). This is because it takes time for the accumulation of misfolded proinsulin in the ER to lead to β-cell apoptosis for patients with heterozygous mutations, while for homozygous *INS* mutations, the production of insulin has been interrupted in the early stages of fetal development. The insulin and C-peptide levels of the proband approached 0 after birth, and the birth weight was only 1.8 kg, which was consistent with the pathogenesis. Within 2 years of birth, his height and weight caught up with his peer group (**Figures 2B, C**), and his motor and intellectual development were no different from those of the same age. Compared with NDM caused by *KCNJ11* and *GATA4* mutations, NDM caused by *INS* mutations has a significantly better prognosis (16). Often, *INS* mutations manifest as low birth weight and ketoacidosis caused by poor blood glucose control. However, as long as the blood glucose level is strictly controlled to avoid ketoacidosis, postnatal growth can reach a normal weight level, and the prognosis is relatively good.

Although the family members with heterozygous mutations still have functional proinsulin synthesis, which can meet the body’s needs through compensation, it is inevitable that such heterozygous mutants are at a higher risk of developing adult-type diabetes or presenting with hyperglycemia compared with healthy people (17).

Currently, insulin injection is the main treatment for NDM worldwide; lifelong insulin injection is considered the only effective treatment, especially for NDM patients with an *INS* mutation. Oral sulfonylureas have been tried to treat NDM patients with an *INS* mutation. However, patients were not sensitive to sulfonylureas, and there are even reported cases of insulin resistance after transformation with sulfonylureas (18). In the animal trials of *INS* mutation-induced diabetes, early insulin treatment was shown to protect mice against insulin resistance, α-cell hyperfunction, β-cell loss, and non-β-cell hyperplasia, which seems to give a significantly better prognosis than treating with oral sulfonylureas (19).

Most patients with PNDM have a degree of intrauterine retarded growth caused by the lack of insulin, so stabilizing the blood glucose is particularly important for catch-up growth in the first 2 years of life. Compared to older patients, children under 2 years of age eat more frequently and irregularly, which makes blood sugar control more difficult. Some studies have suggested that continuous subcutaneous insulin infusion (CSII) can more stably maintain blood sugar and improve the quality of life of infants and their parents, making it a good option for families with patients younger than 2 years old. However, through communication with the family, we know that the use of CSII is not recommended by them as it can affect a child’s sports activities at school, potentially affecting their mental health. In our case, the proband was given protamine biosynthetic human insulin injection (premix 30R) before every meal, and the time between meals was kept as consistent as possible. In addition, he was taught good dietary and exercise habits, had regular outpatient follow-ups, and his dose of insulin was adjusted depending on weight. Moreover, his mother learned to adjust the insulin dosage on a small scale according to the daily diet and exercise; this kind of slight adjustment to daily life is not satisfied by CSII.

In addition, the family members and the child also expressed hope for the development of longer-lasting insulin which could stabilize blood glucose by a once-a-week or even once-a-month injection. Non-invasive insulin delivery (i.e., needle-free injecting) would also considerably improve quality of life. Although there is no obvious effective alternative to lifelong insulin therapy, research at the cellular and molecular level can still provide us with new prospects for the treatment of NDM. Prolactin can protect β-cells from ER stress through the JAK2/STAT5 pathway (20), transcription factors such as GLIS3 can enhance *INS* gene expression (21), rapamycin can improve diabetes and further down-regulate β-cell apoptosis (22), and induced pluripotent stem cells can promote β-cell regeneration (23). The in-depth research on the pathogenesis of NDM, popularization of gene detection technology, and development of new treatment technologies at the molecular and cellular level are still our common goals for the diagnosis and treatment of the disease, and we expect these patients to have a higher quality of life in the future.

In our case, we could not carry out the function verification, another downstream potential start codon is too close making it difficult to get an accurate result from western blotting. We hope to conduct in-depth analysis of protein structure and use other methods in the near future.

## 5 Conclusions

In conclusion, we report a case of NDM caused by a novel *INS* mutation (c.1T>C), expanding the *INS* gene mutation spectrum. The good follow-up prognosis of this case over the past 10 years can bring confidence to the families of NDM patients who need long-term insulin injections. It is clear that in addition to insulin injections, long-term regular follow-up, close monitoring of blood glucose, balanced exercise and diet, and mutual family support are the key factors for a good prognosis.

## Data availability statement

The datasets for this article are not publicly available due to concerns regarding participant/patient anonymity. Requests to access the datasets should be directed to the corresponding author.

## Ethics statement

The studies involving human participants were reviewed and approved by Medical Ethics Committee, West China Second Hospital, Sichuan University. Written informed consent to participate in this study was provided by the participants’ legal guardian/next of kin.

## Author contributions

All the authors have contributed significantly. MT and YF analyzed and interpreted the clinical and biochemical aspects of the patients’ data. YL and MT analyzed and interpreted the molecular genetic data. YF and HW designed the study. MT wrote the manuscript. HW supervised the study and corrected the manuscript. All authors contributed to the article and approved the submitted version.
